# Pile driving repeatedly impacts the giant scallop (*Placopecten magellanicus*)

**DOI:** 10.1038/s41598-022-19838-6

**Published:** 2022-09-13

**Authors:** Youenn Jézéquel, Seth Cones, Frants H. Jensen, Hannah Brewer, John Collins, T. Aran Mooney

**Affiliations:** 1 Biology Department, Woods Hole Oceanographic Institution, Woods Hole, MA USA; 2 MIT-WHOI Joint Program in Oceanography/Applied Ocean Science and Engineering, Cambridge, Woods Hole, MA USA; 3 Department of Biology, Syracuse University, 107 College Place, Syracuse, NY 13244 USA; 4 Geology and Geophysics Department, Woods Hole Oceanographic Institution, Woods Hole, MA USA

**Keywords:** Ecology, Behavioural ecology, Climate-change ecology

## Abstract

Large-scale offshore wind farms are a critical component of the worldwide climate strategy. However, their developments have been opposed by the fishing industry because of concerns regarding the impacts of pile driving vibrations during constructions on commercially important marine invertebrates, including bivalves. Using field-based daily exposure, we showed that pile driving induced repeated valve closures in different scallop life stages, with particularly stronger effects for juveniles. Scallops showed no acclimatization to repetitive pile driving across and within days, yet quickly returned to their initial behavioral baselines after vibration-cessation. While vibration sensitivity was consistent, daily pile driving did not disrupt scallop circadian rhythm, but suggests serious impacts at night when valve openings are greater. Overall, our results show distance and temporal patterns can support future mitigation strategies but also highlight concerns regarding the larger impact ranges of impending widespread offshore wind farm constructions on scallop populations.

## Introduction

Anthropogenic noise is now recognized as a major source of underwater pollution threatening marine animals^1^. Among these sources, pile driving (PD) is associated with the construction of docks, platforms and offshore wind farms (OSW) and is of major concern primarily due to the repeated, high-intensity impulsive noise generated underwater^2–4^. Several studies have described the various impacts of PD on many marine taxa, ranging from temporary changes in behavior to mortality^5–7^. However, the impacts of substrate-borne vibrations from hammering piles into the seabed remain poorly understood^8–10^. This benthic component travels farther and faster than the water-borne signal generating concerns for benthic taxa^8^.

Marine invertebrates are increasingly recognized as sound-sensitive and thus to anthropogenic noise. However, they are also correspondingly data-deficient with respect to underwater noise impacts, a key information gap given their oft-central role in ecosystems and fisheries^11^. Among marine invertebrates, bivalves can detect both water- and substrate-borne vibrations through their abdominal sense organs and statocysts^8^. Bivalves may be particularly vulnerable to vibrations as their benthic and sessile juvenile and adult stages leave little capacity to relocate away from the source. Recent studies showed bivalves respond to low frequencies (i.e., < 1 kHz) by closing their valves^12^. However, the translation of these laboratory experimental results into the field are not yet clear, especially when considering the differing intensities as well as the spatial and temporal scales of potential impacts by anthropogenic activities^10^. Currently, only one field study has described noise impacts on adult scallops. The authors showed that deep-sea oil and gas seismic surveys disrupt scallop metabolism and behaviors^13^. Despite these initial findings and concerns, little is known about responses to younger and highly sensitive stages^10^.

Bivalves are key species in marine ecosystems when considering their high bio deposition rates which enhance natural sedimentation and lessen ecological consequences of eutrophication^14^. In addition, they represent a commercially important taxon, contributing mean annual landings exceeding 15 million tons and worth 20 billion USD^15^. Given the rapid proliferation of anthropogenic activities at sea^1,10^, being able to quantify the associated noise impacts on these ecologically and commercially important species will be critical for the next decade.

**Figure 1:**
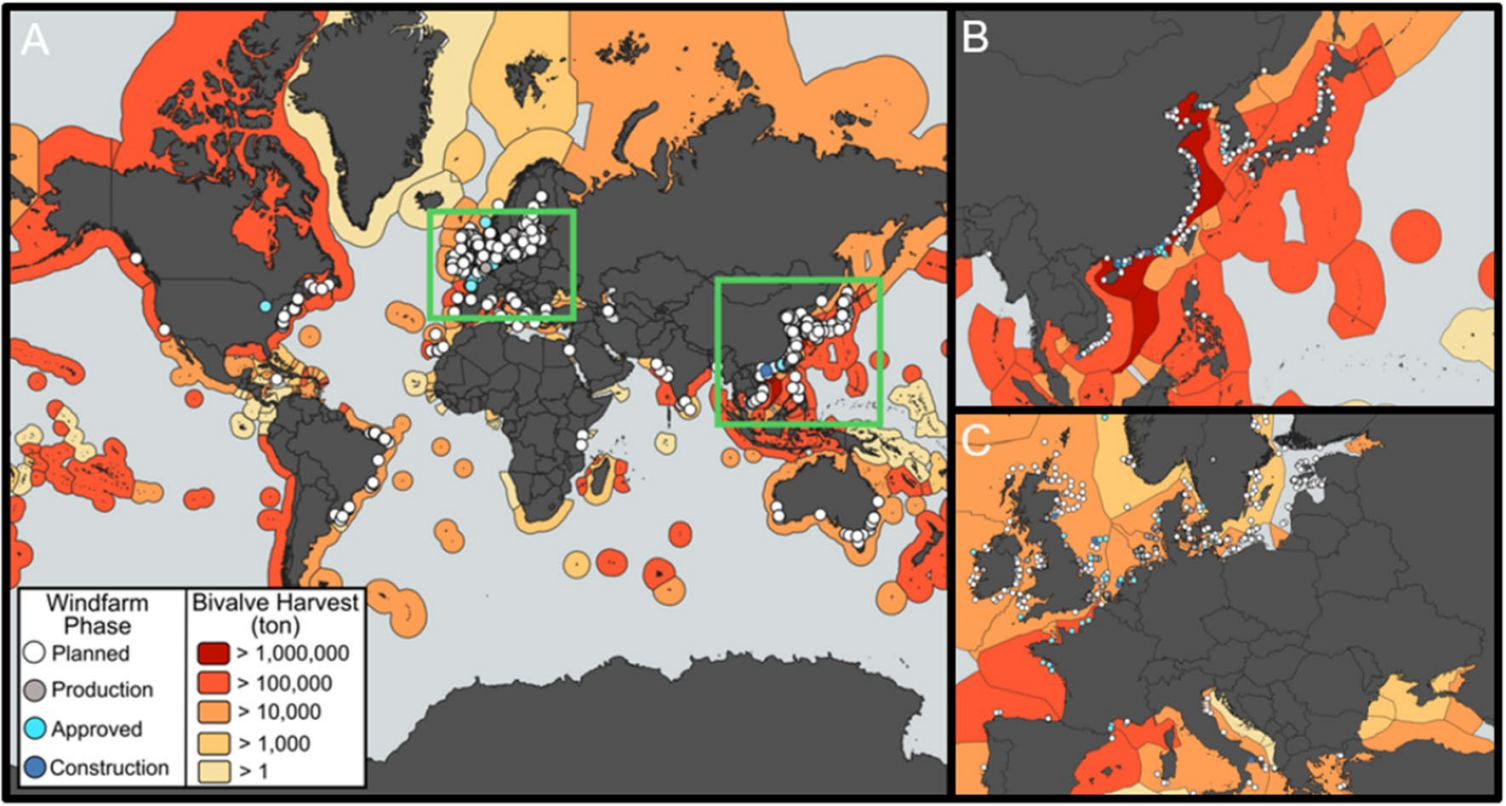
Increasing offshore wind farm constructions overlap with commercial bivalve production worldwide. (**A**) Worldwide estimated offshore wind farm projects and bivalve production in 2022 and 2020, respectively. Offshore wind farm data were retrieved from The Wind Power database (https://www.thewindpower.net/). Bivalve capture and aquaculture fishery production data were sourced from^20^. Green rectangles highlight western Pacific (**B**) and European (**C**) coastal waters. The map was performed in^21^.

Offshore wind farm constructions are increasing dramatically worldwide as nations seek to meet national and global climate targets as well as decrease reliance on fossil fuels (Fig. 1A). As an example, planned OSW projects will increase by 400% within the next decade (The Wind Power database, https://www.thewindpower.net). To support wind farm turbine implementations, the occurrence rates and spatial range of PD are expected to expand globally with many of these projects planned for coastal US, Chinese and European waters (Fig. 1B and C)^16–18^. Consequently, there are considerable overlaps in vital bivalve habitats, fishing zones, and planned OSW development, generating strong conflicts across users of marine resources^19^. There is thus a crucial need to understand the potential impacts of OSW constructions on this taxon^10^.

Here, we used a field-based approach integrating cameras and long-term biologging tags to quantify the behavioral impacts of in situ PD exposure on the giant scallop (*Placopecten magellanicus*). Scallops were exposed daily to repeated PD (30 cm diameter, 10 m long steel monopile) designed to replicate the construction of an OSW at a reasonable experimental scale. We quantified behavioral reactions (partial closure, swimming, total closure) from three different scallop life stages (juveniles, subadults and adults) to a traditional impact hammer (IH; i.e., impulsive signals) as well as a more recently developed vibratory hammer (VH; i.e., continuous signals). Responses were measured at two spatial scales (near site < 10 m vs. far site at 50 m from the pile), and over different (sub second to weeks) time scales, following a before-during-after gradient design.

## Results

### substrate-borne vibrations from pile driving

**Figure 2:**
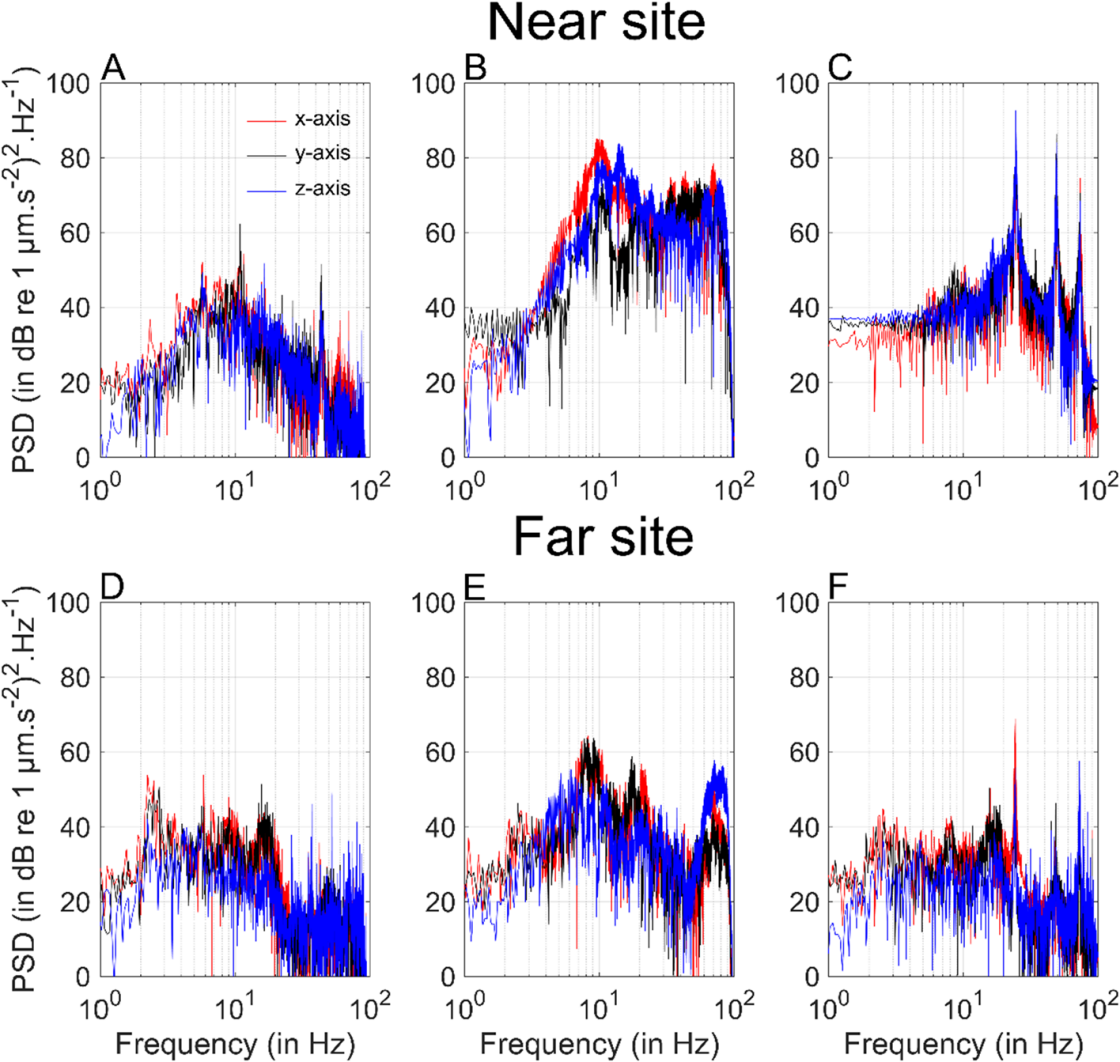
Power spectral densities (PSD) from the temporal series shown in Fig. S1 and S2 at the near (top) and far (bottom) sites without pile driving (**A** and **D**), during the impact (**B** and **E**) and vibratory (C and F) hammers. Colors represent the different recording axes (x: red, y: black, z: blue).

**Figure 3:**
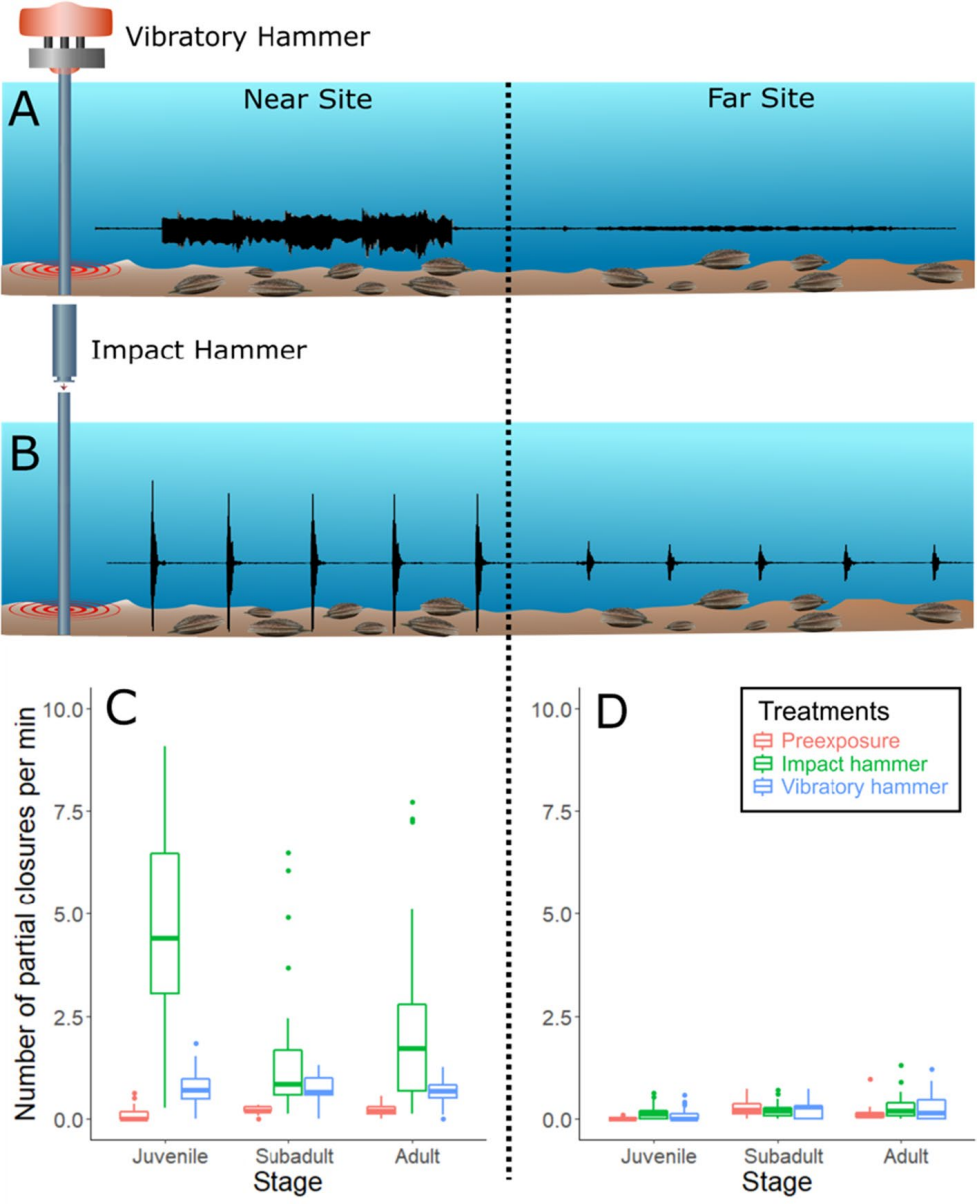
Pile driving induces behavioral responses in nearby scallops across all life stages. Top: experimental set-up showing a typical pile driving event used in this study, starting with the vibratory (**A**) and ending with the impact (**B**) hammers, lasting 30 min in total. Between 4 and 5 piles were successively driven per experimental day. Black waveforms highlight differences in signal types (i.e., **A**: continuous vs. **B**: impulsive) and amplitudes perceived by scallops at both sites. Bottom: response rates of scallops at near (**C**) and far (**D**) sites from the PD for all three tested stages (juveniles, subadults and adults) before PD occurred (i.e., preexposure, in red), during the impact (green) and vibratory (blue) hammer sequences.

Vibrations recorded in absence of PD were low and similar at both study sites (53.86 ± 1.81 and 53.15 ± 5.62 dB re 1 µm·s^-2^). The IH signals were transient pulses (Figs. S1 and S2), and seabed vibration levels ranged from 87.08 ± 2.88 to 109.95 ± 1.25 dB re 1 µm·s^-2^ at the far and near sites, respectively (Table 1). In marked contrast, the VH generated continuous vibrations measured were 62.55 ± 0.60 dB re 1 µm·s^-2^ at 50 m and 86.00 ± 0.98 dB re 1 µm·s^-2^ at 8 m (Figs. S1 and S2). The single strike exposure levels were higher for the VH (near site = 136.60 ± 4.98 dB re (1 µm·s^-2^)^2^.s, far site = 116.20 ± 4.03 dB re (1 µm·s^-2^)^2^.s) compared to the IH (near site = 94.39 ± 1.34 dB re (1 µm·s^-2^)^2^.s, far site = 72.48 ± 2.51 dB re (1 µm·s^-2^)^2^.s; Table 1). The main energy for the IH was dominated by lower frequencies centered at 10 Hz while the VH had peak energies between 10 and 100 Hz (Fig. 2). Overall, seabed vibration levels during PD were high at the near site, while low at the far site (Fig. 3A and B).

**Table 1:** Substrate-borne vibrations’ features from the impact hammer recorded during PD with the geophone at 8 and 50 m calculated on 536 and 315 strikes, corresponding to two days of experiments.

Distance (m)	Inter-pulse interval (s)	Rise time (s)	Acceleration peaks (dB re 1 µm·s^-2^)	Single strike exposure (dB re (1 µm·s^-2^)^2^·s )	Cumulative strike exposure (dB re (1 µm·s^-2^)^2^·s )
8	6.63 ± 0.61	0.08 ± 0.03	109.95 ± 1.25	94.39 ± 1.34	115.67 ± 0.65
50	6.94 ± 1.02	0.08 ± 0.01	87.08 ± 2.88	72.48 ± 2.51	93.13 ± 1.82

Cumulative strive exposure levels at 8 and 50 m were calculated from, on average, 115 and 129 strikes, respectively. Values represent mean ± standard deviation.

### Video observations

A total of 113 different scallops were observed across the two experimental sites (near site: adults = 14, subadults *n* = 16, juveniles = 29; far site: adults = 17, subadults = 18, juveniles = 19) over two consecutive PD events, covering two impact (IH1 and IH2) and two vibratory (VH1 and VH2) hammer exposures.

Scallops located at the near site produced significantly more closures compared to the far site (linear mixed model [LMM]: F_1,119.46_ = 93.565, *p* < 0.001; Fig. 3C and D). In contrast, there were no significant differences in closures for all individuals between preexposure and PD treatments at the far site (Tukey test; preexposure vs. IH1 *p* = 0.15, preexposure versus IH2 *p* = 0.36, preexposure vs. VH1 *p* = 0.71, preexposure vs. VH2 *p* = 0.69). Overall, the mean number of closures at the far site was low for all scallop stages (i.e., < 0.3 closures per min) and closures were not synchronized with IH strikes (see movie S1). Taken together, these results show that scallops were not affected by PD at the far site.

At the near site, PD induced significantly more closures for all scallop life stages compared to preexposure conditions [adults (LMM: F_4,66.441_ = 11.207, *p* < 0.001); subadults (LMM: F_4,60.054_ = 6.191, *p* < 0.001); juveniles (LMM: F_4,72_ = 50.306, *p* < 0.001)] (Fig. 3C). All closures occurred in synchronization with the IH strikes (see movie S2). Closure rates were similar for repeated pile driving events on same day (Tukey test; IH1 vs. IH2 *p* = 0.06, VH1 vs. VH2 *p* = 1), indicating no short-term acclimatization across consecutive PD events. This lack of acclimatization was found for all three life stages (Table S1). Juveniles showed significantly more PD-induced closures compared to subadults and adults (Tukey test; juveniles vs. adults *p* < 0.001, juveniles vs. subadults *p* < 0.01, adults vs. subadults *p* = 0.66) (Fig. 3C). This was exhibited in a mean closure response rate to more than 50% of the total number of the IH strikes (i.e., 5.1 ± 1.9 per min; Fig. 3C). In marked contrast, subadult and adult responded to IH strikes at significantly lower rates (1.5 ± 1.0 and 2.2 ± 1.5 per min, respectively). Finally, all scallop stages at the near site produced significantly more closures when exposed to the IH compared to the VH (Tukey test; adults: IH vs. VH *p* < 0.001, juveniles: IH vs. VH *p* < 0.001, subadults: IH vs. VH *p* < 0.01) (Fig. 3C).

Coughing, a natural behavior used by scallops to oxygenate and clear their pallial cavities, was altered by PD. Scallops coughed significantly more at the far site (LMM: F_1,109.39_ = 24.088, *p* < 0.001). At both sites, juveniles coughed significantly more compared to adults and subadults (Tukey test; far site: juveniles vs. adults *p* < 0.001, juveniles vs. subadults *p* < 0.001; near site: juveniles vs. adults *p* < 0.001, juveniles vs. subadults *p* < 0.05), suggesting a relative higher activity rate. Yet, juveniles produced significantly fewer coughing events at the near site (LMM: F_1,45.444_ = 36.34, *p* < 0.001), suggesting that PD exposure disrupted this behavior. Finally, we did not observe any scallop performing swimming behaviors in the video recordings throughout the entire experimental period. Total closures occurred in only 8 individuals (juveniles = 26%, subadults = 11%, adults = 18%). These animals were all located at the near site and lasted between 0.12 and 2.51 min in duration.

### Valve angles

**Figure 4:**
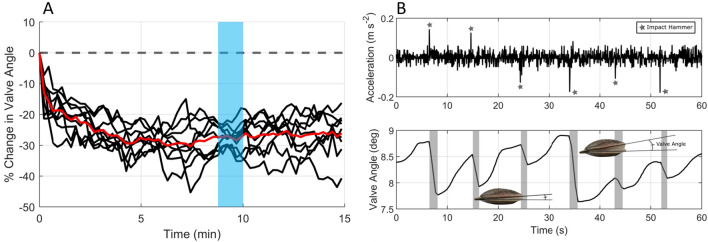
Scallops decrease valve angle during pile driving events, with no acclimation over long (i.e., days) and short (i.e., minutes to hours) time. Responses (valve angle, in degrees) from tagged scallops to PD at two different time scales at the near site: (**A**) Mean changes in valve angle in response to daily PD (black lines) compared to the preexposure sequence (dashed line). The red line highlights the mean of 9 days of PD exposure. Note the quick and dramatic decrease in valve angle during the first minute of PD exposure. Blue area covers the time period highlighted in B. (**B**) Synchronized data from one tagged scallop placed at the near site, with the accelerometer (top) and magnetometer (bottom). Each strike from the impact hammer (stars) was detected by the tagged scallop, which responded by partially closing its valves (shaded area).

All tagged scallops at the near site showed valve closures in response to PD, resulting in a reduction of 30% in valve angle compared to pre-exposure (Fig. 4). In marked contrast, no closures were observed in direct response to PD for tagged scallops at the far site. Indeed, there were no significant differences in valve angles across the different exposure treatments (i.e., preexposure, exposure, recovery) at the far site (LMM: F_2,602.09_ = 0.946, *p* = 0.39).

The LMMs showed that only the addition of exposure (compiling both daily IH and VH treatments) significantly improved the model fit (see Table S2), indicating both PD types were the impetus for this difference. Hence, the mean valve angles were significantly lower during IH and VH exposure compared to preexposure periods (5.52 ± 1.75° vs. 7.34 ± 2.33° respectively; Tukey test, *p* < 0.001). In addition, there were no significant differences in valve angles between preexposure and postexposure (i.e., recovery) sequences (Tukey test, *p* = 0.44), demonstrating that scallops returned to their initial valve angle baselines shortly (within 15 min) after PD exposure. Finally, there was no significant effect of day of exposure on valve angle (LMM: F_1,7.132_ = 1.42, *p* = 0.27), showing that scallops did not habituate to PD throughout the two weeks of exposure (see Fig. 4A).

**Figure 5:**
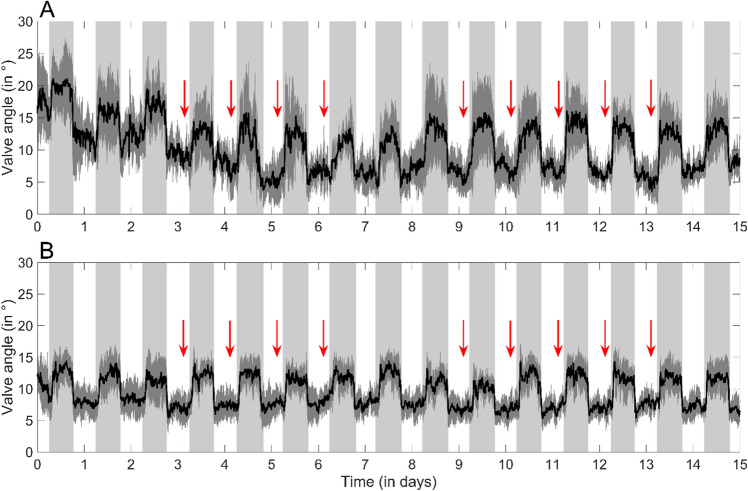
Scallop diurnal rhythm, with higher valve angles during the night, may render these bivalves more sensitive to night-time pile driving events. Valve angles (in degrees) from tagged subadult scallops (*n* = 10) recorded at the two experimental sites (**A**: near site; **B**: far site) across two weeks of PD exposures, represented as means (bold black lines) and standard deviations (dark gray). White and shaded areas indicate daytime and nighttime periods (respectively) over the 15-d monitoring period (11–26th September 2021). Red vertical arrows highlight the time when daily PD occurred.

All tagged scallops exhibited a maximum valve opening behavior at night and a minimum opening during daytime at both experimental sites (Fig. 5), with a dominant periodicity of τ = 24.0 h (g.test, *p* < 0.001; Fig. S3). Thus, while short term responses were repeated within and between days, daily PD exposure did not modify the scallops’ general circadian rhythm.

## Discussion

To the authors knowledge, these data provide the first field-based evidence of substrate-borne vibrations associated with pile driving impacting a commercially and ecologically vital marine invertebrate, inducing repeated behavioral changes with likely energetic consequences. Responses (i.e., partial valve closures) were seen across all three scallop life-stages examined and juveniles appeared to be most sensitive. Further, responses were dose (i.e., range) dependent and were not observed at a more distant site (i.e., 50 m). Importantly, scallops did not show short-term (within days) and long-term (across days) habituation to pile driving events, suggesting extended PD exposure can have cumulative energetic reactions on scallops. Overall, these diverse and repeated results across life-stages, with particular influence on young animals, indicate that OSW construction activities have the potential to repeatedly and consistently impact scallops.

A common criticism of previous noise exposure experiments (including PD for marine invertebrates) is that studies mostly occurred in the laboratory where it is challenging to recreate realistic sound exposure levels, rending their findings difficult to translate to real-life scenarios^10^. To circumvent this issue and more accurately recreate the noise field associated with OSW constructions, we exposed scallops to actual PD in a coastal environment. However, it is notable that wind turbine foundations are many times larger (often 10 m diameter and 150 m long monopiles)^22^ than the 0.3 m diameter, 10 m height steel monopile used here. For example, the IH used in our study generated substrate-borne vibrations at 8 m from the pile that were similar in amplitude to the recordings made at hundreds of meters from an OSW construction in the North Sea using a 6 m diameter, 55 m long monopile^23^. Hence, we caution that the short spatial scale behavioral responses observed for scallops in our study are likely to occur over much higher distances during OSW constructions. Considering the distances between wind turbine foundations (~ 1 km), our results imply OSW constructions have the potential to impact scallops at the population level.

Juveniles produced significantly more valve closures when exposed to PD compared to subadults and adults (Fig. 3C), underscoring the general concern that early life stages in marine invertebrates are particularly sensitive to environmental stressors^24^. Overall, juveniles responded to more than 50% of the IH strikes over 15 min periods (~ 110 strikes per event). This trend was repeated for each IH sequence showing they did not habituate to the noise exposure. In addition, PD disrupted their classic coughing behavior, which is commonly used by scallops to oxygenate their mantle cavity. When considering their daily rapid growth^25^, longer-term PD exposure could lead in stunted growth, as has been highlighted for *P. maximus* juveniles in response to hypoxia and toxic phytoplankton^26^. Limited stunted growth and the resulting size reduction have previously been shown to influence both the population dynamics of scallops and socio-economics of their fisheries^27^.

Interestingly, the IH impacted the scallops significantly more than the VH, indicating that impulsive signals, because of their high peak particle motion and short rise, induce greater harm than continuous and lower intensity signals (Fig. 3C). While these abrupt onset signals may be more deleterious, we do not know yet how the shape of these signals affects response rates. For example, further from the source we would expect not only a decrease in noise amplitude, but also an increase in rise time, making a more gradual onset. One or both factors could play a role in how these animals respond to this acoustic stressor^28^. Yet, this result underscores the importance of taking into account the type of exposure signal when assessing impact studies on marine animals. Overall, previous studies also found that impact PD noise leads to more stressful behaviors compared to continuous noise in fish^29–31^ although it is less clear in previous laboratory studies with marine invertebrates^32^. Hence, we suggest the vibratory technique can be used as a mitigation strategy to reduce impacts on scallop populations during OSW constructions.

We did not find any significant differences in either closure rates or valve angles of scallops to repeated PD sequences (Fig. 4). This indicates that scallops did not acclimatize to both short (3 h) and long (2 weeks) term repetitive PD. This is vastly different from laboratory studies on fish and marine invertebrates that reported apparent habituation to impulsive noise^7,31,33^. In this context, the duration and timing of marine OSW constructions are important considerations when assessing impacts. Indeed, year-long projects, typical for many construction activities, would have substantial energetic effects in scallops.

Pile driving exposure did not result in energetically expensive behaviors such as swimming. This result is fully coherent with the only other field-based noise study where adult scallops *P. fumatus* were exposed to seismic air gun signals^13^. This may be because this behavior is energetically costly^34^, or the acoustic stressor may not be aversive enough to induce a flight-based response. However, all scallops reacted to PD at the near site by partially or fully closing their valves (Figs. 3 and 4), which is a common response to environmental changes such as the presence of a nearby predator^35^. The energetic and ecological consequences of observed partial valve closures are not yet clear.

Previous studies showed scallops undertake infrequent short-duration, active behaviors such as valve closures, but these behaviors still induce large energetic costs^34,36^. Immediately after movements, scallops increase oxygen uptake by opening more their valves to recover their energy debt^34^. Scallops exhibiting excess post-exercise oxygen consumption will be limited in their ability to swim away from predators or dig themselves into sediment^34^. Thus, PD inducing valve closures at an increased rate could lead scallops to be more vulnerable to predation. We did not find any significant differences in the valve angles of scallops between the preexposure and postexposure periods over two weeks of PD exposure. This result shows that scallops recovered quickly (i.e., within 15 min), similar to *P. fumatus* after seismic exposure^13^. However, longer-term PD exposures during OSW constructions may result in extend fatigue, rending scallop populations more vulnerable to predation over time.

Finally, all tagged scallops opened their valves in a diurnal circadian pattern (τ = 24.0 h), where they exhibited maximal valve opening during nighttime and minimum opening during daytime hours (Fig. 5). This observation, new to scallops, is coherent with previous laboratory and field studies for other bivalves, such as mussels^37–39^ and oysters^40^. Scallops possess a complex visual system with up to 200 eyes that respond to dark, moving features^41^. This spatial vision is mostly used for detecting nearby predators^42^. Hence, the light—dark cycle represents an environmental cue entraining an endogenous gaping rhythm in these bivalves, as hypothesized previously in mussels and oysters^37,43^. The adaptive significance of this nocturnal gaping is a strategy to feed while minimizing the likelihood of predation^37^.

In our study, we found a significant reduction in valve angle during daytime PD when scallops were not fully open (Figs. 4 and 5), thus responses were inherently milder than may be seen at night. This result raises clear concerns about the stronger impacts that PD could have on scallops during the night. OSW constructions intensify worldwide and developers are increasingly seeking permission to conduct PD at night to speed constructions. Hence, management agencies should take into account how such changes might increase impacts on nocturnally filter-feeding and economically bivalves.

## Conclusion

Evidence of diverse impacts from both anthropogenic noise and substrate-borne vibrations is mounting, underscoring the need for broader understanding of noise impacts on marine organisms to inform management across taxonomic groups and life-stages. This field-based study highlighted the importance of considering the behavioral, and potentially physiological, impacts of PD on bivalves when designing OSW plans. The persistent in situ impacts to a range of life-stages are concerning. Yet our data show there may be means to mitigate these impacts, including distance (i.e., pile driving far away from scallop populations to avoid behavioural responses), received levels and adopting other PD techniques such as the vibratory hammer. Further, for animals showing diel patterns such as scallops, impacts may vary accordingly between day and night. Understanding such diurnal rhythms, and how impacts change as a consequence, are critical to plan and mitigate impacts.

Although this is the first experiment assessing the impacts of PD in a marine invertebrate under real-time conditions, further studies are now required to assess the responses of wild scallop populations at the scale of real OSW constructions^10^. The present study can be used as a blueprint where similar methods can be applied in close coordination with developers to assess dose–response curves for commercially important bivalves as OSW constructions expand. The mechanistic underpinnings and ecological impacts of these responses to PD exposure require further characterization to understand the overall economic and ecological implications. To avoid present and future conflicts between fishermen, policy makers and OSW developers, a comprehensive understanding of PD impacts will be required in order to facilitate effective management.

## Materials and methods

### Animal collection, characteristics and holding conditions

This study was performed using three different scallop sizes reflecting contrasting life stages. 60 adults (shell diameter = 11.5 ± 0.9 cm; mean ± s.d.) caught via dredging off George’s Bank were acquired from fishermen in New Bedford (USA) on the 17th of August and 9th of September 2021. We also purchased 80 subadults (7.1 ± 1 cm) and 100 juveniles (3.0 ± 0.1 cm) at the Pine Point Oyster Company (Cape Elizabeth, USA) on the 2nd of September 2021. After collection, all animals were transferred to the facilities of the Woods Hole Oceanographic Institution (WHOI) where they were acclimatized in tanks. Adults and subadults were uniquely numbered to recognize individuals throughout the experiment.

### Location and characteristics of the pile driving

Pile driving experiments took place from the 14th to 29th of September 2021 in an open area off WHOI’s pier (41.52° N, 70.67° W). It is a shallow water habitat with 3–5 m depth during low and high tide (respectively), with a flat bottom composed of homogeneous sand and silt. OSW foundations primarily consist of a mono-cylindrical steel structure driven into the seabed using hydraulic or diesel hammers that employ impact or vibratory PD^4^. For the purpose of this study, we simulated the construction of a wind turbine with a cylindrical steel monopile (length: 10.0 m, diameter: 0.3 m, thickness: 0.02 m) with a steel plate welded at the bottom that was impact driven 2.5 m off a dock. A crane (American 595) with a 20 m long boom lifted a VH (weight: 212 kg, H&M model 135) that was first used to secure the pile into the seabed at a rate of 1150 blows per min. Then, a steel IH (weight: 1500 kg) was manually dropped at a height of 1.2 m and released against the top of the pile at a rate of 8 to 12 strikes per min for a duration of ~ 15 min (see movie S3). The energy generated by the impact was ~ 16 kJ per strike. Once the pile was at a depth of up to 5 m below the water–sediment interface, the VH was used again to remove the pile and drive it at another location adjacent to the previous hole. Hence, one PD event comprised a VH phase followed by an IH phase, separated by five minutes from each other, and was repeated four to five times for each exposure day.

### Recording devices

#### Seabed vibrations

A WHOI short-period ocean bottom seismometer (OBS) was used to record substrate-borne vibrations from PD over a 9-day time period of this experiment. A calibrated geophone (model GS-11D from Geospace^®^) with a 200 Hz frequency sampling was buried in the seafloor by divers at 1 m from scallop cages. It recorded ground-motion along three-orthogonal axes (one vertical and two horizontal). All data were recorded on a Quanterra Q330 24-bit data-logger after × 30 pre-amplification. Recordings were made at 8 and 50 m from the PD.

#### Short term behaviors: videos

Individual scallop behaviors were recorded every day using 8 GoPro HERO6 black cameras mounted onto a 0.5 kg lead placed inside the four cages. Recordings started 1 min after the cameras’ insertion in scallop cages and lasted ~ 90 min, which usually covered two consecutive PD sequences. Cameras were placed in front of different individuals each day. Scallop behaviors were annotated in BORIS (v7.12.2)^44^ and included coughing, partial closure, total closure and swimming^34,35^.

#### Long term behaviors: tags

We measured valve angles from 14 subadult scallops using small Axy 5 XS bio-loggers (Technosmart Europe srl, www.technosmart.eu). These tags contain both an accelerometer and a magnetometer, the latter being used for measuring valve gap, which were glued on the dorsal valves, and a magnet was located on the ventral valves. Leveraging the magnetometers proportional sensing of magnetic field strength, the valve angles were determined from the tag outputs^37,40^. The magnetometer’s voltage (in mV) was recorded at a frequency of 2 Hz. The accelerometer recorded acceleration in three orthogonal axes with a sampling frequency of 25 Hz, and was used to record seabed vibrations arising from PD events which permitted us to synchronize them with valve angles. At the end of the experiments, tag data were retrieved and calibrated to compute the relationship between magnetometer outputs and scallop valve angles using the same procedure described in previous studies^37,40^. In total, 10 tagged scallops (near site: *n* = 4; far site: *n* = 6) were analyzed for this study. They recorded valve angles for a period of two weeks, covering 9 days of PD exposure.

#### Environmental parameters

Seawater temperature and light were measured continuously every 5 min during the experiments using HOBO Pendant G data logger (UA-004–64, Onset Computer Corporation). The recorded seawater temperature ranged from 22.4 ± 0.1 °C to 20.9 ± 0.1 °C at the start and at the end of the experimental period, respectively. It is to note that the seawater temperature was close to the upper limit of *P. magellanicus* thermal tolerance (although wild populations in shallow bays have been found to face temperatures above 20 °C)^45^, which could have affected their physiological metabolism^46^. However, all tagged scallops showed a clear natural circadian rhythm over the two experimental weeks (see results). Such a result highlights that they were well acclimatized to their environment and exhibited natural behaviors during PD exposure.

### Experimental procedure

We built an experiment following a before-during-after gradient designed across two different distances from the PD, which is recommended to study the impacts of OSW constructions^47^. Equal groups of scallops were placed in one of two types of acoustically transparent, custom sized-cages. Adults and subadults were situated into two large rectangular cages (4.0 × 3.0 × 1.0 m) constructed with plastic 1-cm mesh netting encasing dock wooden piles. Juveniles were placed in two small rectangular cages (0.6 × 0.4 × 0.2 m) created using polypropylene plastic boxes covered with 2-mm plastic mesh netting. The cages were placed at two distances (near site < 10 m and far site = 50 m) from the PD (see Fig. S4).The bottom was not meshed to allow scallops to be in contact with the substrate. The cages were used to both control the distances of the scallops from PD and to isolate them from predators. After the initial acclimation period we placed 30 adults, 30 subadults (including tagged scallops) and 40 juveniles in each cage, where they were held for a 3-d acclimation period before the experiment started. Scallops were exposed daily (except on weekends) to four to five consecutive PD events, which lasted ca. two hours total each afternoon, starting at ca. 1:30 pm every day.

### Seabed vibrations

Custom analysis scripts in MATLAB R2021a (Cambridge, USA) were used to evaluate various metrics from the PD substrate-borne vibrations recorded by the OBS. We quantified these metrics over two different PD signals: the classic IH strikes and the VH. For the IH strikes, we calculated the pulse duration (in ms) as the duration each signal took to rise from 5 to 90% of its maximum absolute value of acceleration, as well as the rise time (in ms) where each signal reached from 5% energy to peak^48^. The energy arising from the strikes was described with the 0-peak acceleration levels (in dB re 1 µm·s^-2^), the single strike exposure levels (SEL_ss_; in dB re (1 µm·s^-2^)^2^.s) using the pulse duration containing 90% of the pulse energy, and the cumulative strike exposure level over a PD event (SEL_cum_; in dB re (1 µm·s^-2^)^2^.s). Because VH generated continuous signals, we reported here the root-mean-square of the recorded acceleration in the 90% energy window (in dB re 1 µm·s^-2^)^49^ as well as the SEL_ss_.

### Statistical analyses

Statistical significance for all tests was set at 0.05. Reported values represent means ± SEM (standard error of the mean).

#### Video data

Behavioral analysis across the different PD treatments was quantified as the numbers of behavioral events per minute. We decided to focus our analysis in coughing and partial closures as they were the most common observed behaviors performed by all scallops (see Results). Partial closures related to fish swimming close to the cage (i.e., visible on videos) were removed from the analysis. Differences amongst the PD treatments were assessed with linear mixed models using the “lmer” function in the “lme4” package available in R^50^. Sites (near and far), scallop stages (juvenile, subadult and adult) and PD treatments (preexposure, IH1, VH1, IH2, VH2) were included as categorical explanatory variables, while the effect of different individuals was modelled as a random effect on the intercept. We also performed pairwise tests to compare PD treatments using post hoc Tukey tests with the “emmeans” package^51^. All analyses were performed in R (version 3.6.3).

#### Tag data

We performed a linear mixed-effects analysis between scallop valve gap angle and different fixed effects. Data were log transformed to normally distribute the residuals, which was further confirmed through the visual examination of residual plots. As a hierarchical model selection method, we generated five nested models: a null model with only random effects (i.e. individual and date) and four extending models where each new model was identical to the previous one except for the addition of a fixed effect^52^. Fixed effects included preexposure vs. exposure vs. postexposure (with exposure compiling all IH and VH sequences), exposure type (i.e. IH vs. VH sequences), and exposure sequence (i.e. each isolated IH vs. VH sequence). A stepwise likelihood ratio test was performed to evaluate the importance of each of the fixed effects and to determine which model was best fitted to the data. Finally, we compared the mean valve gap angles between preexposure and post exposure periods (15 min each) to assess whether scallops returned to their initial valve angle baselines after PD exposure.

We also used a periodogram analysis to identify the dominant frequency in time series measurements over 15 days of recordings, which is a method applied to data when periods are not known a priori^53^. The largest periodogram value corresponds to the period that explains the largest proportion of the variance in the time series. The statistical significance of the largest periodogram value for each individual was verified using the Fisher's *g*-statistic^54^ using custom-made scripts in MATLAB (version R2021a).

### Ethical statement

Experiments with scallops are not subject to restriction for animal scientific research according to the USA legislation.

## Supplementary Information

Supplementary Information.

## Data Availability

All data used for the analyses are available on request to the corresponding author.

## References

[1] Duarte CM, Chapuis L, Collin SP, Costa DP, Devassy RP, Eguiluz VM, Erbe C, Gordon TA, Halpern BS, Harding HR, Havlik MN (2021). The soundscape of the Anthropocene ocean. Science.

[2] Bailey H, Brookes KL, Thompson PM (2014). Assessing environmental impacts of offshore wind farms: Lessons learned and recommendations for the future. Aquat. Biosyst..

[3] Dahl PH, de Jong CA, Popper AN (2015). The underwater sound field from impact pile driving and its potential effects on marine life. Acoust. Today..

[4] Mooney TA, Andersson MH, Stanley J (2020). Acoustic impacts of offshore wind energy on fishery resources. Oceanography.

[5] Madsen PT, Wahlberg M, Tougaard J, Lucke K, Tyack AP (2006). Wind turbine underwater noise and marine mammals: implications of current knowledge and data needs. Mar. Ecol. Prog. Ser..

[6] Slabbekoorn H, Bouton N, Van Opzeeland I, Coers A, Ten Cate C, Popper AN (2010). A noisy spring: the impact of globally rising underwater sound levels on fish. Trends Ecol. Evol..

[7] Jones IT, Stanley JA, Mooney TA (2020). Impulsive pile driving noise elicits alarm responses in squid (*Doryteuthis pealeii*). Mar. Pollut. Bull..

[8] Roberts L, Elliott M (2017). Good or bad vibrations? Impacts of anthropogenic vibration on the marine epibenthos. Sci. Total. Environ..

[9] Hawkins AD, Hazelwood RA, Popper AN, Macey PC (2021). Substrate vibrations and their potential effects upon fishes and invertebrates. J. Acoust. Soc. Am..

[10] Popper AN, Hice-Dunton L, Jenkins E, Higgs DM, Krebs J, Mooney A, Rice A, Roberts L, Thomsen F, Vigness-Raposa K, Zeddies D (2022). Offshore wind energy development: Research priorities for sound and vibration effects on fishes and aquatic invertebrates. J. Acoust. Soc. Am..

[11] Williams R, Wright AJ, Ashe E, Blight LK, Bruintjes R, Canessa R, Clark CW, Cullis-Suzuki S, Dakin DT, Erbe C, Hammond PS (2015). Impacts of anthropogenic noise on marine life: Publication patterns, new discoveries, and future directions in research and management. Ocean. Coast. Manag..

[12] Roberts L, Cheesman S, Breithaupt T, Elliott M (2015). Sensitivity of the mussel *Mytilus edulis* to substrate-borne vibration in relation to anthropogenically generated noise. Mar. Ecol. Prog. Ser..

[13] Day RD, McCauley RD, Fitzgibbon QP, Hartmann K, Semmens JM (2017). Exposure to seismic air gun signals causes physiological harm and alters behavior in the scallop *Pecten fumatus*. Proc. Natl. Acad. Sci..

[14] Newell RI (2004). Ecosystem influences of natural and cultivated populations of suspension-feeding bivalve molluscs: a review. J. Shellfish. Res..

[15] Wijsman, J.W.M., Troost, K., Fang, J. & Roncarati, A. Global production of marine bivalves. Trends and challenges. *Goods and services of marine bivalves*, (Eds. Small, A.D., Ferrerira, J.G., Grant, J., Petersen, J.K., Strand, O.) 7–26 (Springer, Cham, 2019).

[16] Perveen R, Kishor N, Mohanty SR (2014). Off-shore wind farm development: Present status and challenges. Renew. Sust. Energ. Rev..

[17] Vaissière AC, Levrel H, Pioch S, Carlier A (2014). Biodiversity offsets for offshore wind farm projects: The current situation in Europe. Mar. Policy..

[18] Musial, W.D., Beiter, P.C., Spitsen, P., Nunemaker, J. & Gevorgian, V. 2018 offshore wind technologies market report. US Department of Energy (2019).

[19] Lacroix D, Pioch S (2011). The multi-use in wind farm projects: more conflicts or a win-win opportunity?. Aquat. Living. Resour..

[20] FishstatJ. FishStatJ-Software for Fishery and Aquaculture Statistical Time Series. *FAO Fisheries Division [online]*, *Rome*. Accessed April 10, 2022. (2020).

[21] Flanders Marine Institute. Maritime Boundaries Geodatabase: Maritime Boundaries and Exclusive Economic Zones (200NM), version 11. Available online at https://www.marineregions.org/ (2019).

[22] Kallehave D, Byrne BW, LeBlanc Thilsted C, Mikkelsen KK (2015). Optimization of monopiles for offshore wind turbines. Philos. Trans. R. Soc. A.

[23] Bruns, B., Stein, P., Kuhn, C., Sychla, H. & Gattermann, J. Hydro sound measurements during the installation of large diameter offshore piles using combinations of independent noise mitigation systems. *Proceedings of the Inter-noise Conference* 1–10 (Melbourne, Australia, 2014).

[24] Hunt HL, Scheibling RE (1997). Role of early post-settlement mortality in recruitment of benthic marine invertebrates. Mar. Ecol. Prog. Ser..

[25] Pilditch CA, Grant J (1999). Effect of variations in flow velocity and phytoplankton concentration on sea scallop (*Placopecten magellanicus*) grazing rates. J. Exp. Mar. Biol. Ecol..

[26] Chauvaud L, Thouzeau G, Paulet YM (1998). Effects of environmental factors on the daily growth rate of *Pecten maximus* juveniles in the Bay of Brest (France). J. Exp. Mar. Biol. Ecol..

[27] Rheuban JE, Doney SC, Cooley SR, Hart DR (2018). Projected impacts of future climate change, ocean acidification, and management on the US Atlantic Sea scallop (*Placopecten magellanicus*) fishery. PLoS ONE.

[28] Hawkins AD, Pembroke AE, Popper AN (2015). Information gaps in understanding the effects of noise on fishes and invertebrates. Rev. Fish. Biol. Fish..

[29] Neo YY, Seitz J, Kastelein RA, Winter HV, Ten Cate C, Slabbekoorn H (2014). Temporal structure of sound affects behavioural recovery from noise impact in European seabass. Biol. Conserv..

[30] Sabet SS, Neo YY, Slabbekoorn H (2015). The effect of temporal variation in sound exposure on swimming and foraging behaviour of captive zebrafish. Anim. Behav..

[31] Radford AN, Lèbre L, Lecaillon G, Nedelec SL, Simpson SD (2016). Repeated exposure reduces the response to impulsive noise in European seabass. Glob. Change. Biol..

[32] Solan M, Hauton C, Godbold JA, Wood CL, Leighton TG, White P (2016). Anthropogenic sources of underwater sound can modify how sediment-dwelling invertebrates mediate ecosystem properties. Sci. Rep..

[33] Hubert J, Booms E, Witbaard R, Slabbekoorn H (2022). Responsiveness and habituation to repeated sound exposures and pulse trains in blue mussels. J. Exp. Mar. Biol. Ecol..

[34] Robson AA, Chauvaud L, Wilson RP, Halsey LG (2012). Small actions, big costs: the behavioural energetics of a commercially important invertebrate. J. R. Soc. Interface..

[35] Thomas GE, Gruffydd LD (1971). The types of escape reactions elicited in the scallop *Pecten maximus* by selected sea-star species. Mar. Biol..

[36] Livingstone DR, Dezwaan A, Thompson RJ (1981). Aerobic metabolism octopine production and phosphoarginine as sources of energy in the phasic and catch adductor muscles of the giant scallop *Placopecten magellanicus* during swimming and the subsequent recovery period. Comp. Biochem. Physiol. B. Biochem. Mol. Biol..

[37] Comeau LA, Babarro JM, Longa A, Padin XA (2018). Valve-gaping behavior of raft-cultivated mussels in the Ría de Arousa Spain. Aquac. Rep..

[38] Wilson R, Reuter P, Wahl M (2005). Muscling in on mussels: new insights into bivalve behaviour using vertebrate remote-sensing technology. Mar. Biol..

[39] Comeau LA, Babarro JM (2014). Narrow valve gaping in the invasive mussel *Limnoperna securis*: implications for competition with the indigenous mussel *Mytilus galloprovincialis* in NW Spain. Aquac. Int..

[40] Comeau LA, Mayrand E, Mallet A (2012). Winter quiescence and spring awakening of the Eastern oyster *Crassostrea virginica* at its northernmost distribution limit. Mar. Biol..

[41] Palmer BA, Taylor GJ, Brumfeld V, Gur D, Shemesh M, Elad N, Osherov A, Oron D, Weiner S, Addadi L (2017). The image-forming mirror in the eye of the scallop. Science.

[42] Chappell DR, Horan TM, Speiser DI (2021). Panoramic spatial vision in the bay scallop *Argopecten irradians*. Proc. R. Soc. B..

[43] Mat AM, Massabuau JC, Ciret P, Tran D (2012). Evidence for a plastic dual circadian rhythm in the oyster *Crassostrea gigas*. Chronobiol. Int..

[44] Friard O, Gamba M (2016). BORIS: a free, versatile open-source event-logging software for video/audio coding and live observations. Methods. Ecol. Evol..

[45] Dickie LM, Medcof JC (1963). Causes of mass mortalities of scallops (*Placopecten magellanicus*) in the southwestern Gulf of St Lawrence. J. Fish. Res. Board. Can..

[46] Coleman S, Cleaver C, Morse D, Brady DC, Kiffney T (2021). The coupled effects of stocking density and temperature on Sea Scallop (*Placopecten magellanicus*) growth in suspended culture. Aquac. Rep..

[47] Methratta ET (2020). Monitoring fisheries resources at offshore wind farms: BACI vs. BAG designs. ICES. J. Mar. Sci..

[48] ISO, 18406. Underwater acoustics measurement of radiated underwater sound from percussive pile driving. *International Organization for Standardization* (Geneva, Switzerland), 1–33 (2017).

[49] Madsen PT (2005). Marine mammals and noise: Problems with root mean square sound pressure levels for transients. J. Acoust. Soc. Am..

[50] Bates D, Mächler M, Bolker B, Walker S (2015). Fitting linear mixed-effects models using lme4. J. Stat. Softw..

[51] Lenth, R.V. emmeans: Estimated marginal means, aka least squares means. R package version 1.3.5.1. Retrieved from http://CRAN.R-project.org/package=emmeans (2019).

[52] Kragh IM, McHugh K, Wells RS, Sayigh LS, Janik VM, Tyack PL, Jensen FH (2019). Signal-specific amplitude adjustment to noise in common bottlenose dolphins (*Tursiops truncatus*). J. Exp. Biol..

[53] Warner RM (1998). Spectral Analysis of Time-Series Data.

[54] Fisher RA (1929). Tests of significance in harmonic analysis. Proc. Math. Phys. Eng. Sci..

