# Supplementary material for: Pile driving repeatedly impacts the giant scallop (Placopecten magellanicus)
Source: Sci Rep. 2022 Sep 13;12:15380. doi: 10.1038/s41598-022-19838-6 (PMC9470578; doi:10.1038/s41598-022-19838-6)
Supplement: Supplementary file 1 — Supplementary Information. [file 41598_2022_19838_MOESM1_ESM.docx]

**SUPPLEMENTARY MATERIALS**

Table S1. Outputs from linear mixed models the number of partial closures per min. and stages across the different pile driving categories (IH = impact hammer, VH = vibratory hammer) at 10 m:

| Stage | Fixed effect | Effect size ± SE | Post hoc comparison | Tuckey test |
| --- | --- | --- | --- | --- |
| Adults | LMM: F_4,66.441_ = 11.207, p < 0.001 | | | |
|  | Intercept (Control) | 0.21 ± 0.31 | Control – IH1 | < 0.001 |
|  | IH1 | 2.12 ± 0.39 | Control – IH2 | < 0.001 |
|  | IH2 | 1.80 ± 0.39 | Control – VH1 | 0.74 |
|  | VH1 | 0.48 ± 0.39 | Control – VH2 | 0.86 |
|  | VH2 | 0.40 ± 0.41 | IH1 – IH2 | 0.93 |
|  |  |  | IH1 – VH1 | < 0.001 |
|  |  |  | IH1 – VH2 | < 0.001 |
|  |  |  | IH2 – VH1 | < 0.05 |
|  |  |  | IH2 – VH2 | < 0.01 |
|  |  |  | VH1 – VH2 | 0.99 |
| Subadults | LMM: F_4,60.054_ = 6.191, p < 0.001 | | | |
|  | Intercept (Control) | 0.21 ± 0.25 | Control – IH1 | < 0.001 |
|  | IH1 | 1.48 ± 0.32 | Control – IH2 | < 0.05 |
|  | IH2 | 1.02 ± 0.32 | Control – VH1 | 0.57 |
|  | VH1 | 0.48 ± 0.32 | Control – VH2 | 0.44 |
|  | VH2 | 0.60 ± 0.35 | IH1 – IH2 | 0.59 |
|  |  |  | IH1 – VH1 | < 0.05 |
|  |  |  | IH1 – VH2 | 0.11 |
|  |  |  | IH2 – VH1 | 0.44 |
|  |  |  | IH2 – VH2 | 0.77 |
|  |  |  | VH1 – VH2 | 0.99 |
| Juveniles | LMM: χ F_4,72_ = 50.306, p < 0.001 | | | |
|  | Intercept (Control) | 0.14 ± 0.35 | Control – IH1 | < 0.001 |
|  | IH1 | 5.00 ± 0.44 | Control – IH2 | < 0.001 |
|  | IH2 | 3.71 ± 0.44 | Control – VH1 | 0.66 |
|  | VH1 | 0.60 ± 0.44 | Control – VH2 | 0.63 |
|  | VH2 | 0.62 ± 0.44 | IH1 – IH2 | < 0.05 |
|  |  |  | IH1 – VH1 | < 0.001 |
|  |  |  | IH1 – VH2 | < 0.001 |
|  |  |  | IH2 – VH1 | < 0.001 |
|  |  |  | IH2 – VH2 | < 0.001 |
|  |  |  | VH1 – VH2 | 1 |

Table S2: Model selection using likelihood-ratio tests with valve angle (VA) recorded from tagged subadult scallops at less than 10 m from the PD. Four linear mixed-effects models (fixed effects in bold, random effects in italics) were tested hierarchically, with χ² and P-values for each model representing a test against model one level down. The results show that only the addition of Exposure (E, regrouping impact and vibratory hammer sequences) significantly improved the Null model. ET = Exposure type (impact vs. vibratory hammer), ES = Exposure Sequence (isolated impact and vibratory hammer sequences across one PD event).

| Model | | d.f. | ∆AIC | ∆BIC | χ² | P-value |
| --- | --- | --- | --- | --- | --- | --- |
| Full model | VA~**E+ET+ES+ET×ES**+(*1\|Individual*)+(*1\|Date*) | 312 | 276.78 | 218.24 |  |  |
| Model3 | VA~**E+ET+ES**+(*1\|Individual*)+(*1\|Date*) | 312 | 276.78 | 218.24 | 7.86 | 0.45 |
| Model2 | VA~**E+ET**+(*1\|Individual*)+(*1\|Date*) | 320 | 325.74 | 298.42 | 3.08 | 0.08 |
| Model1 | VA~**E**+(*1\|Individual*)+(*1\|Date*) | 321 | 331.09 | 307.67 | 29.57 | < 0.001 |
| Null model | VA~(*1\|Individual*)+(*1\|Date*) | 4 | 316.43 | 300.82 |  |  |


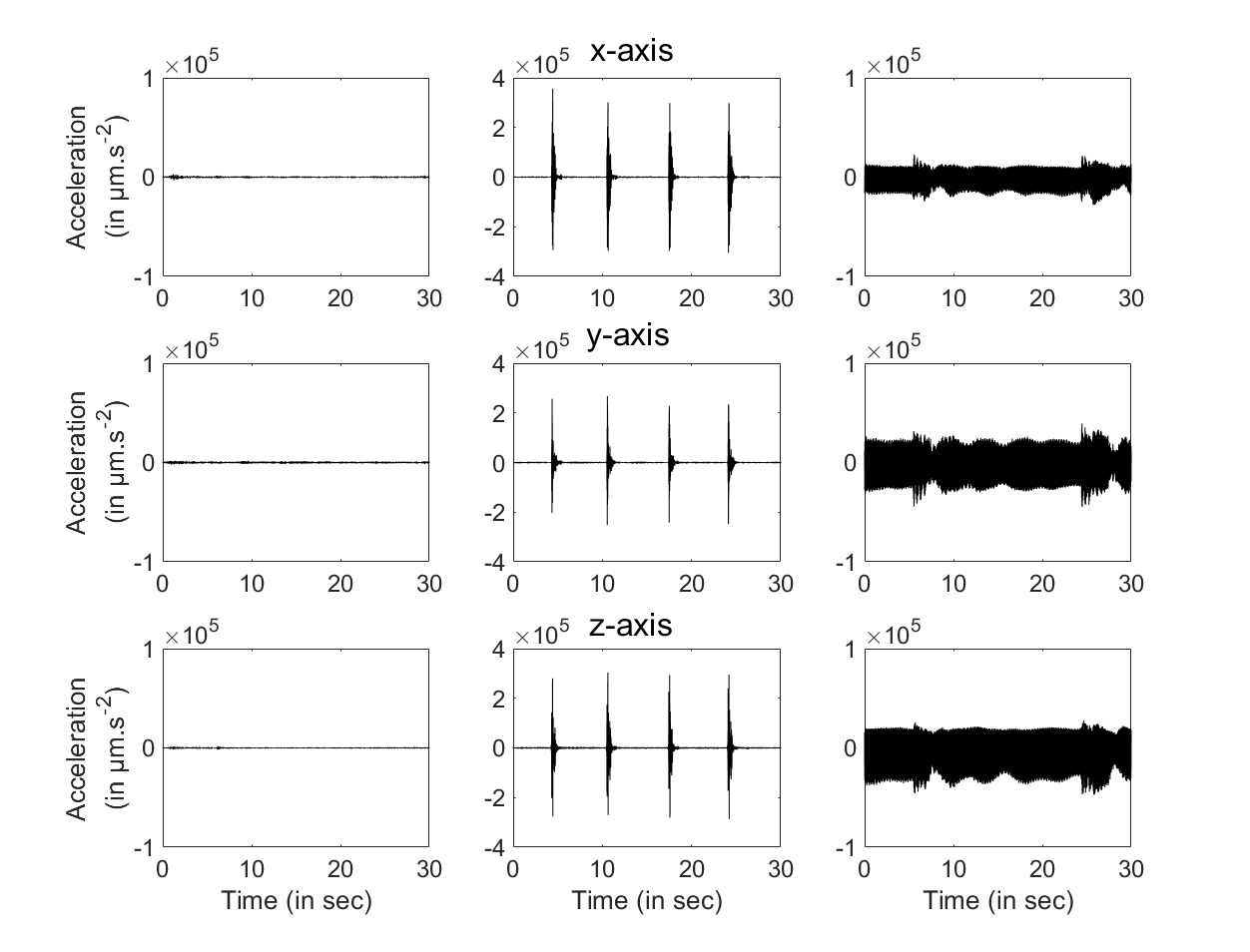


Figure S1. Temporal series of substrate-borne vibrations recorded by the OBS at the near site (8 m from the pile) without pile driving (left), during the impact (middle) and vibratory (right) hammers.


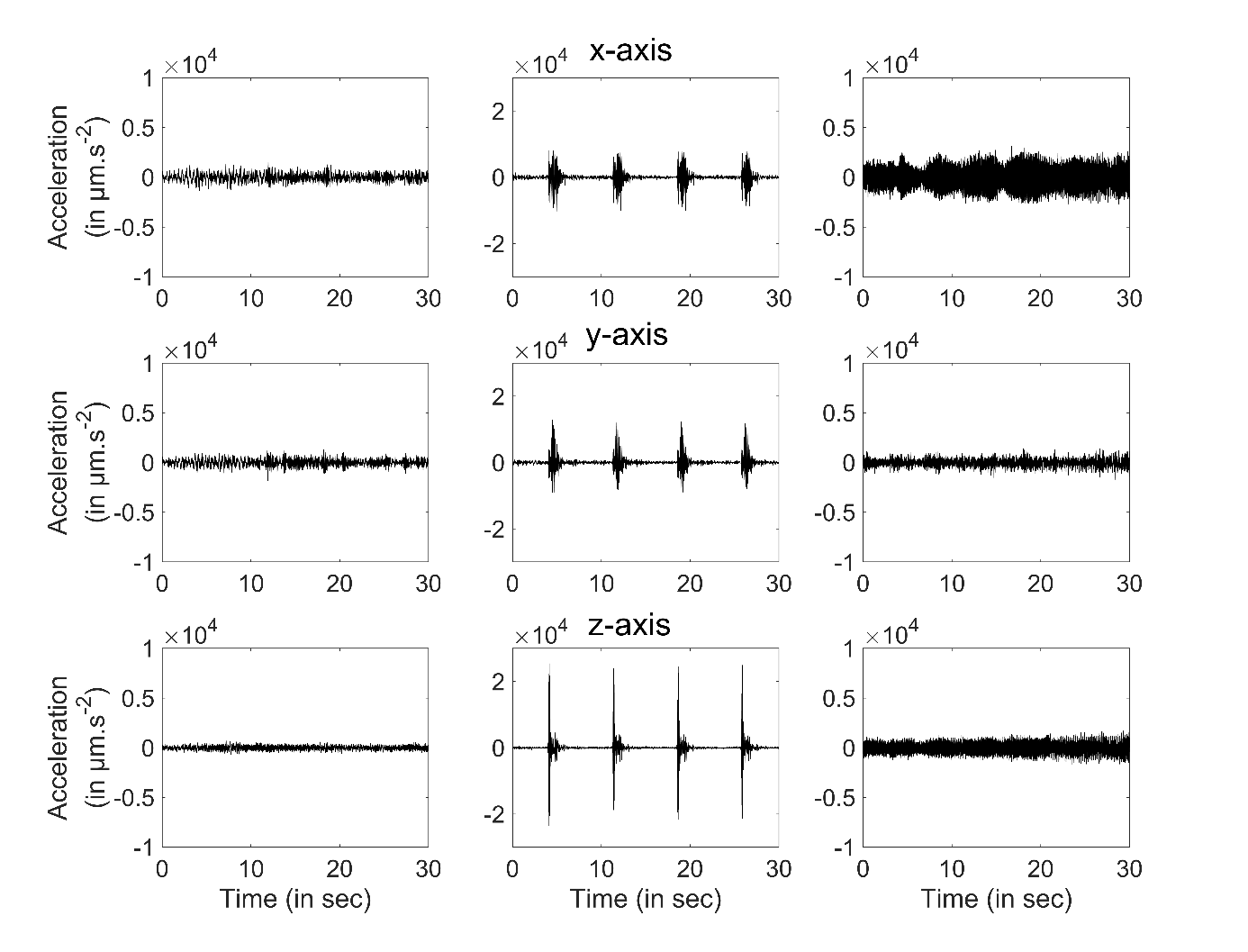
Figure S2. Temporal series of substrate-borne vibrations recorded by the OBS at the far site (50 m from the pile) without pile driving (left), during the impact (middle) and vibratory (right) hammers.


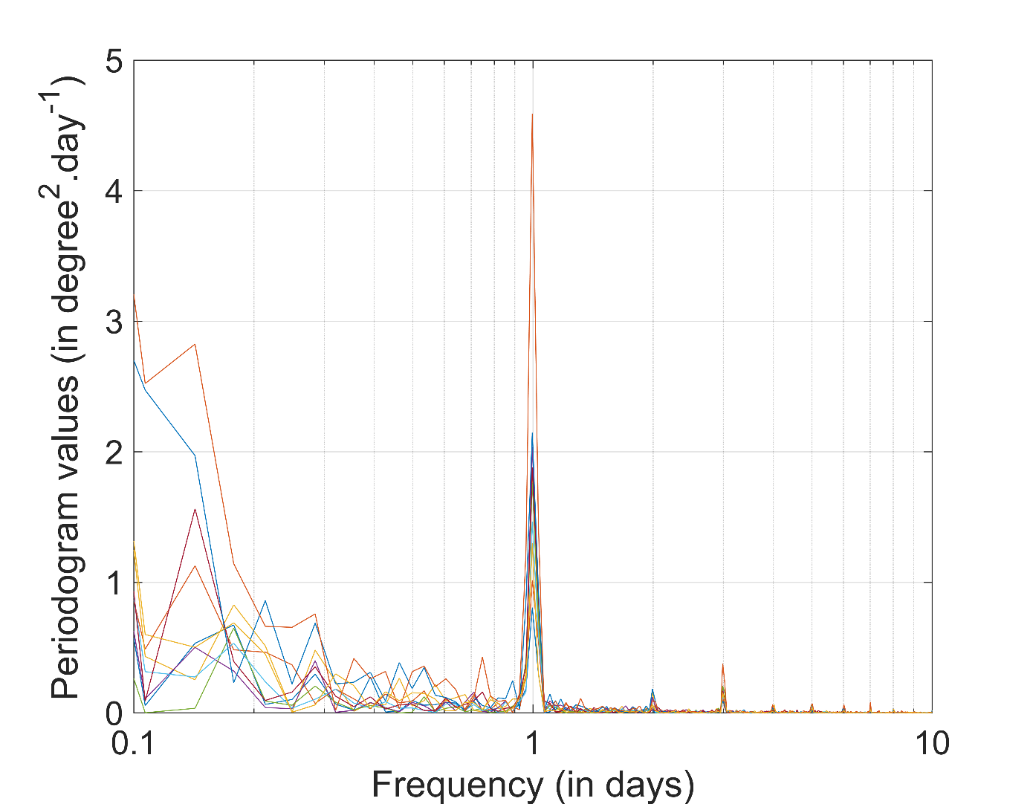


Figure S3. Periodogram values from magnetometer data recorded during two experimental weeks (n = 10 tagged scallops).


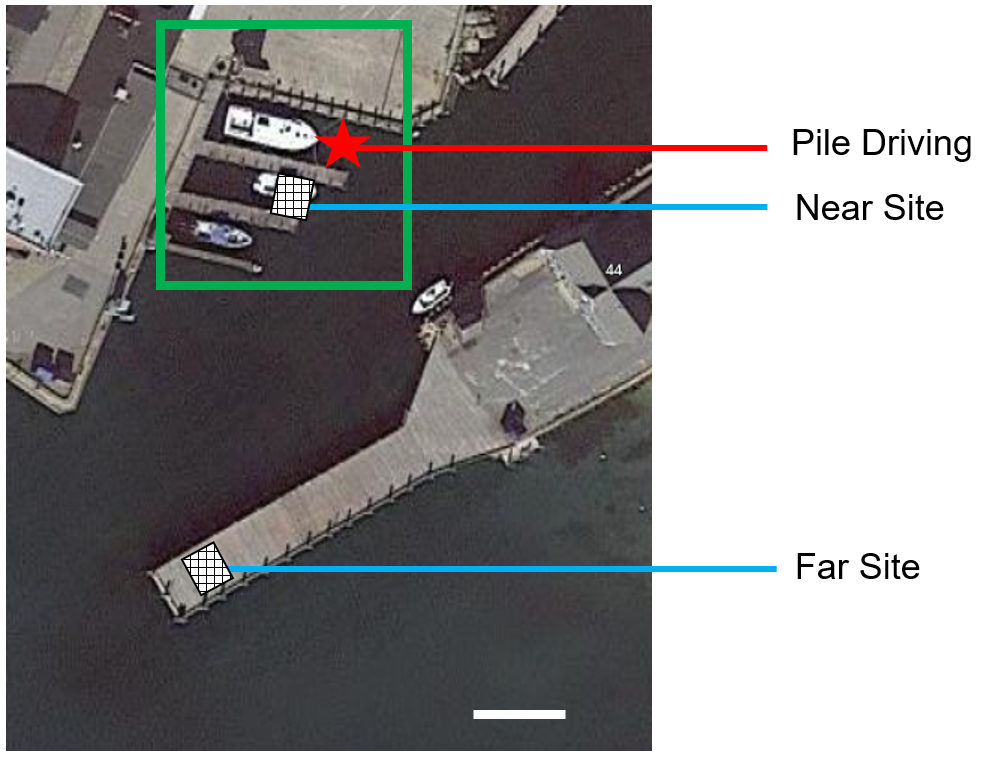


Figure S4. Top view of the experimental site at Woods Hole harbor. White bar is 10 m. The green square represents the area shown by the Movie S1.

Movie S1. All three scallop life stages responded to pile driving by closing their valves at the near site.

Movie S2. At the far site, pile driving did not impact any scallop.

Movie S3. Field view of the pile driving set-up at the near site with a focus on the impact hammer. Note that scallop cages were located between the two docks at less than 10 m from the pile (see fig. S5).
